# A best evidence summary of anticoagulation management for extracorporeal membrane oxygenation in adults

**DOI:** 10.1097/MD.0000000000049542

**Published:** 2026-07-03

**Authors:** Dacai Xu, Lu Wang, Zheng Li, Weifeng Tang

**Affiliations:** a Yancheng No. 1 People’s Hospital, Affiliated Hospital of Medical School, Nanjing University, Yancheng, Jiangsu, China.

**Keywords:** anticoagulation, evidence summary, evidence-based basis, extracorporeal membrane oxygenation

## Abstract

**Background:**

This study aims to synthesize the highest quality evidence for the anticoagulation management of extracorporeal membrane oxygenation (ECMO) in adults, providing an evidence-based foundation for clinical practice in ECMO anticoagulation.

**Methods:**

An evidence summary approach was employed, adhering to the evidence summary reporting standards established by the Evidence-Based Nursing Center of Fudan University. Utilizing the “6S” evidence hierarchy model, comprehensive searches were conducted across both domestic and international databases for evidence pertaining to anticoagulation in ECMO patients. The literature types encompassed guidelines, expert consensus statements, best practices, and systematic reviews. The search period extended from January 1, 2019, to May 31, 2024. Two evidence-based reviewers independently extracted and synthesized relevant data from qualifying literature.

**Results:**

The initial search yielded 553 articles, from which 10 were selected following rigorous screening. These comprised 3 clinical guidelines, 3 clinical decisions, 2 systematic reviews, and 2 expert consensus articles. In total, 30 best evidence statements were distilled, categorized into pre-anticoagulation evaluation, individualized anticoagulation strategies, complication prevention and treatment, and nursing care. Among these, 25 were strong recommendations, 5 were weak recommendations, and 14 were secondary evidence.

**Conclusion:**

The study identified 30 best evidence statements for anticoagulation management in adult ECMO. Given the diverse origins of these evidence statements, clinical practitioners must consider contextual factors such as specific clinical settings and patient conditions prior to application. Future research should focus on the clinical application of this evidence summary to foster evidence implementation and to develop standardized clinical nursing protocols.

## 1. Introduction

Extracorporeal membrane oxygenation (ECMO) is an advanced extracorporeal support device that replicates cardiopulmonary function, providing critical respiratory and circulatory support. This allows the heart and lungs to rest, facilitating the gradual recovery of these organs to normal function.^[1]^ ECMO is increasingly recognized for its efficacy in treating critically ill patients, especially in the context of significant global health crises, such as the novel coronavirus (COVID-19) pandemic.^[2]^ Beyond its established roles in perioperative support for heart and lung transplantation and in the management of severe cardiopulmonary failure, ECMO demonstrates promising applications in conditions like traumatic shock, septic shock, fulminant myocarditis, autoimmune diseases, and non-cardiac surgeries for critically ill patients.^[3]^ Despite its potential, the weaning rate from ECMO remains relatively low at 48%, primarily due to the severity of underlying conditions and complications.^[4]^ Among these, bleeding is the most prevalent and poses a significant challenge, emphasizing the need to reduce bleeding incidence to improve ECMO treatment success rates.^[5]^

During ECMO therapy, the inflammatory response and coagulation system mutually activate and exacerbate each other, disrupting the body’s coagulation-anticoagulation balance. This imbalance heightens the procoagulant state, increasing the risk of thromboembolic events such as stroke, limb ischemia, and necrosis.^[3]^ Consequently, anticoagulation management is crucial during ECMO treatment. However, excessive anticoagulation elevates the risk of bleeding. Data from the Extracorporeal Life Support Organization (ELSO) indicate that 40.2% of patients experienced thrombotic or bleeding complications during ECMO anticoagulation, with the mortality rate from bleeding significantly surpassing that from thrombosis.^[4]^ Additionally, ELSO data over the past 2 decades reveal a bleeding incidence of 30.8%.^[6]^ While advancements in materials and catheterization techniques have reduced bleeding at catheterization sites, other types of bleeding remain unchanged, highlighting the need for improved anticoagulation strategies. Currently, no standardized anticoagulation protocol exists for adult ECMO patients, and practices vary widely. Although ELSO’s 2014 guidelines provide detailed recommendations on anticoagulant selection and monitoring, the complex interplay between ECMO anticoagulation and bleeding complicates the maintenance of an optimal balance between anticoagulation and hemorrhage.^[7]^

Anticoagulation management is a critical and challenging aspect of ECMO therapy, spanning the entire treatment duration. The primary goal of ECMO anticoagulation is to minimize thrombosis while maximizing endogenous procoagulant activity to reduce bleeding.^[8]^ Current research, both domestic and international, predominantly focuses on anticoagulation protocols, the prevention and treatment of adverse reactions, and efficacy evaluations. Despite these efforts, clinical application faces several challenges, including the lack of evidence-based guidelines for pre-anticoagulation risk assessment, individualized anticoagulation strategies, and the prevention, treatment, and nursing of complications. An evidence summary is a research method that systematically retrieves, appraises, and synthesizes existing high-quality evidence, presenting it narratively to form comprehensive evidence items that can guide clinical practice. It differs from standard systematic reviews in that it does not perform a meta-analysis. In response to these gaps, this study adopts an evidence summary approach to synthesize the best evidence for ECMO anticoagulation management across 3 key areas: pre-anticoagulation risk assessment, individualized anticoagulation strategies, and the prevention, treatment, and nursing of complications. The aim is to provide a robust theoretical foundation for clinical practice and promote the effective implementation of evidence-based anticoagulation management in ECMO therapy.

## 2. Methods

### 2.1. Establishing evidence-based questions

The research questions for this study were formulated using the “population, intervention, professionals, outcomes, setting, types of evidence” model.^[9]^ The population (P) comprised ECMO patients; the intervention (I) was anticoagulation; the professionals (P) applying the evidence were clinical medical staff; the outcomes (O) included thrombosis, bleeding, coagulation, limb ischemia, and limb necrosis; the setting (S) for evidence application was the intensive care unit; and the types of evidence (T) reviewed encompassed clinical practice guidelines, evidence summaries, systematic reviews, and expert consensus statements.

### 2.2. Literature search

Utilizing the “6S” evidence model,^[10]^ a thorough search was conducted to identify relevant evidence on anticoagulation management for ECMO patients. The types of literature sought included clinical guidelines, expert consensus statements, best practices, systematic reviews, and evidence summaries.

#### 2.2.1. Search resources

① Chinese Databases: CNKI, Wanfang, VIP Chinese Journals, Yimaitong, among others.
② International Databases: Web of Science, Ovid, PubMed, Cochrane Library, Embase, UpToDate, National Institute of Clinical Medicine, Ontario Registered Nurses Association, International Guidelines Network, American Acute Care Association, ELSO, European Society of Critical Care Medicine, and others.

#### 2.2.2. Search terms

The search terms encompassed extracorporeal oxygenation, extracorporeal life support, artificial lung, anticoagulation, thrombosis, embolism, coagulation, hemorrhage, limb ischemia, and limb necrosis.

#### 2.2.3. Inclusion criteria

① Subjects were ECMO patients aged ≥ 18 years.
② Relevant literature focused on the evaluation, management, prevention, and treatment of complications, as well as nursing practices related to ECMO anticoagulation.
③ Literature types included guidelines, evidence summaries, systematic reviews, expert consensus, clinical decision-making, and original research.
④ Language restrictions were limited to Chinese and English.

#### 2.2.4. Exclusion criteria

① Duplicate articles.
② Articles without full-text access.
③ Drafts, plans, abstracts, and reports.
④ Literature with low-quality evaluations.

#### 2.2.5. Search strategy

The search strategy employed a combination of subject terms and free-text keywords. For instance, in PubMed, the search terms included: (“extracorporeal membrane oxygenation” [Title/Abstract] OR “extracorporeal life support” [Title/Abstract] OR “ECMO” [Title/Abstract] OR “artificial lung” [Title/Abstract]) AND (“anticoagulation” [Title/Abstract] OR “thrombosis” [Title/Abstract] OR “embolism” [Title/Abstract] OR “coagulation” [Title/Abstract] OR “bleeding” [Title/Abstract] OR “limb ischemia” [Title/Abstract] OR “limb necrosis” [Title/Abstract]). The search covered publications from January 1, 2019, to May 31, 2024.

### 2.3. Literature quality evaluation criteria

The quality of guidelines was assessed using the Appraisal of Guidelines for Research and Evaluation II tool,^[11]^ which encompasses 6 domains and twenty-three criteria. Expert consensus statements and systematic reviews were evaluated according to the Joanna Briggs Institute (JBI) Center for Evidence-Based Healthcare Expert Consensus Evaluation Criteria (2016 version),^[12]^ which includes 6 evaluation criteria. Each item was rated by the evaluators as “yes,” “no,” “unclear,” or “not applicable.” Clinical decision-making literature was presumed to have high quality by default, as such documents are typically synthesized and updated by authoritative institutions based on high-quality original research and are recognized as high-level evidence sources in evidence-based methodology.

### 2.4. Quality evaluation process

Two members who had received training and guidance in evidence-based courses independently completed the process according to the above standards and discussed and negotiated when there was a disagreement. If no agreement could be reached, a third party was invited to intervene and arbitrate. When there were conflicting conclusions of evidence from different sources, this study gave priority to the latest published and higher-quality evidence.

### 2.5. Evidence extraction and grading

The extracted evidence items were graded using the JBI evidence pre-grading system (2014 version),^[13,14]^ which classifies evidence into levels 1 to 5, with level 1 being the highest. When the same evidence appeared in multiple documents, the one with the highest evidence level was used. The evidence-based team, comprising one chief physician in critical care medicine, 2 deputy chief nurses, one graduate student, and 2 specialist nurses, further validated the evidence levels based on the JBI FAME (Feasibility, Appropriateness, Meaningfulness, and Effectiveness) framework.^[15]^ The team then assigned recommendation levels, with Level A indicating a strong recommendation and Level B a weak recommendation, based on feasibility, clinical significance, appropriateness, and effectiveness.

### 2.6. Registration

This study was reported in accordance with the evidence summary reporting standards of the Evidence-Based Nursing Center of Fudan University and was registered with the same center under the title “Best Evidence Summary for Anticoagulation Management of Extracorporeal Membrane Oxygenation in Adults” (registration code: ES20245455). PROSPERO registration was not applicable, as this study is an evidence summary rather than a systematic review or meta-analysis; PROSPERO only accepts registrations for systematic reviews with health-related outcome protocols.

### 2.7. Ethical approval

As this study is an evidence summary and did not involve the collection of primary patient data or the implementation of any interventions, ethical approval was not required. According to the institutional guidelines of the Ethics Committee of Yancheng No. 1 People’s Hospital, informed consent was waived for this type of study.

## 3. Results

### 3.1. Literature screening process and results

**Figure 1.**
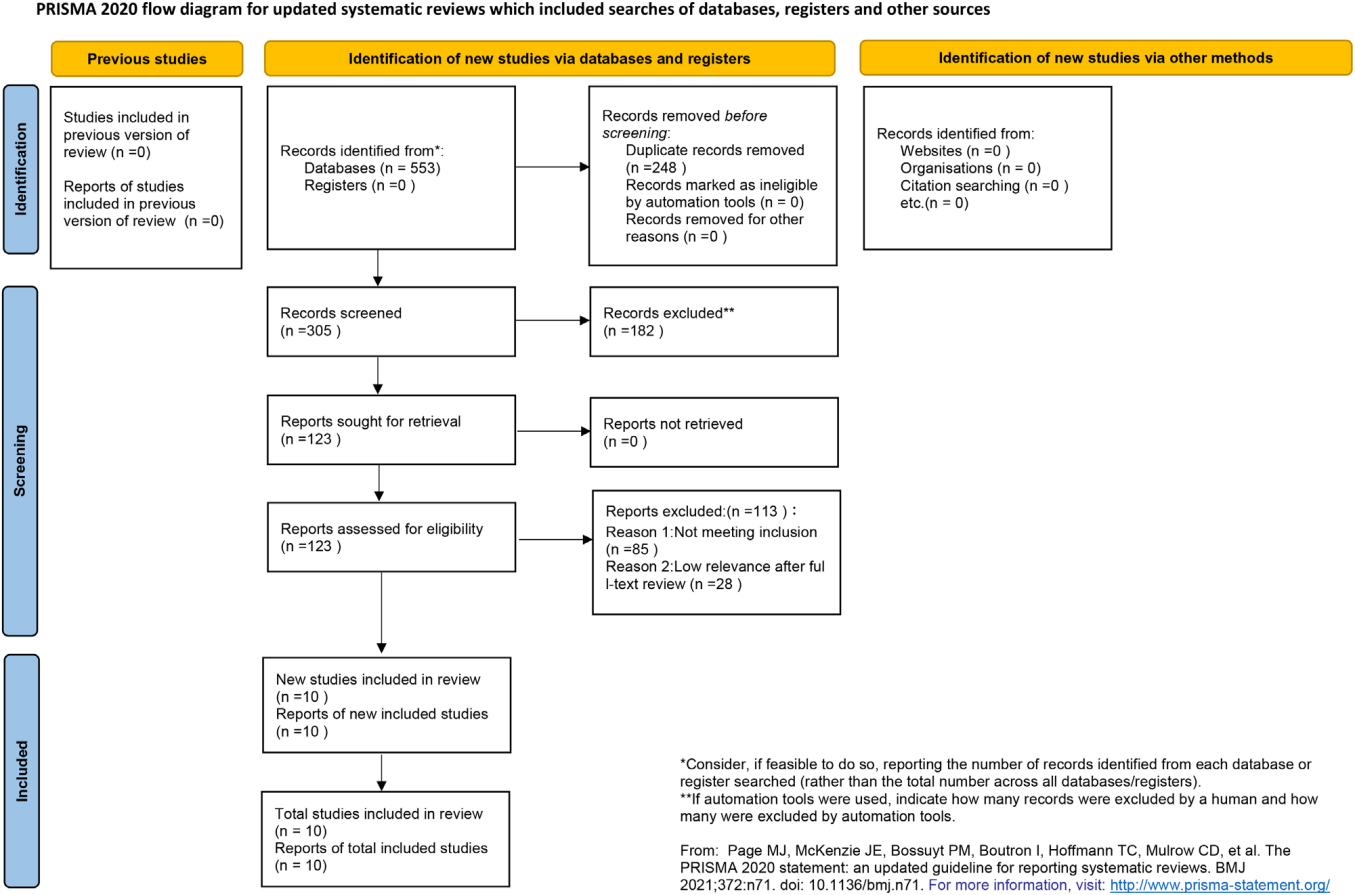
PRISMA 2020 flow diagram of literature screening process.

The initial search yielded 553 articles. After removing 248 duplicates, 182 articles were excluded based on their titles and abstracts as they were irrelevant to the topic. A further 85 articles were discarded for not meeting the inclusion criteria, including: 32 articles focused on pediatric ECMO patients (age < 18 years), 27 articles addressed non-anticoagulation aspects of ECMO management, 15 articles were original research studies that did not provide synthesized evidence, 7 articles were case reports or case series, and 4 articles were letters, editorials, or commentaries. Following full-text review, an additional 28 articles were excluded due to low relevance. Ultimately, 10 articles were included in the analysis^[16–25]^: 3 clinical guidelines,^[17,18,21]^ 3 clinical decision-making articles,^[16,23,24]^ 2 systematic reviews,^[19,20]^ and 2 expert consensus articles.^[22,25]^ The detailed literature screening process is presented in Figure 1, and the basic characteristics of the included literature are summarized in Table 1.

**Table 1:** Basic features of the included literature.

Literature	Published	Literature source	Literature topic	Type of evidence
Brokmeier HM et al^[16]^	2022	PubMed	Anticoagulation management in ECMO	Clinical decision making
Helms J et al^[17]^	2023	PubMed	ECMO Guidelines for Anticoagulation in Adult Patients	Clinical guidelines
McMichael ABV et al^[18]^	2022	ELSO	2021 ELSO Anticoagulation Guidelines for Adults and Children	Clinical guidelines
Hasegawa D et al^[19]^	2023	PubMed	Comparison of heparin and bivalirudin in ECMO anticoagulation	Systematic review
Hla TTW et al^[20]^	2024	NICE	Effectiveness of anti-Xa assay monitoring in venous anticoagulation for extracorporeal membrane pulmonary oxygenation	Systematic review
Tonna JE et al^[21]^	2021	PubMed	Guidelines for Extracorporeal Membrane Pulmonary Oxygenation in Adults	Clinical guidelines
Chinese Society of Cardiothoracic Anesthesiology et al^[22]^	2020	Wanfang Database	Expert Consensus on the Clinical Use of Extracorporeal Membrane Pulmonary Oxygenation in Adults under Different Circumstances (2020 Edition)	Expert consensus
Levy JH et al^[23]^	2022	PubMed	Anticoagulation management during ECMO	Clinical decision making
Stammers AH et al^[24]^	2022	Up To Date	Use of anticoagulants in ECMO	Clinical decision making
Ji Bingyang et al^[25]^	2021	CNKI (China National Knowledge Infrastructure)	2020 EACTS/ELSO/STS/AATS expert consensus on extracorporeal life support after adult cardiac surgery	Expert consensus

ECMO = extracorporeal membrane oxygenation, ELSO = Extracorporeal Life Support Organization.

### 3.2. Quality evaluation of included literature

#### 3.2.1. Quality evaluation of clinical decision-making

The 3 clinical decision-making articles included in this study were presumed to be of high quality and were all recommended for use.

#### 3.2.2. Quality evaluation of guidelines

Three clinical guidelines were evaluated using the Appraisal of Guidelines for Research and Evaluation II instrument. All 3 guidelines achieved high overall quality with standardized scores ≥60% in most domains, and were therefore recommended for clinical application. Detailed results are presented in Table 2.

**Table 2:** AGREE II quality assessment results of included clinical guidelines.

Included literature	Scope and purpose	Stakeholder involvement	Rigor of development	Clarity of presentation	Applicability	Editorial independence	Overall evaluation
Helms J et al^[17]^	89%	78%	94%	92%	67%	83%	Strongly recommended
McMichael ABV et al^[18]^	83%	75%	97%	75%	83%	83%	Strongly recommended
Tonna JE et al^[21]^	94%	25%	100%	88%	94%	25%	Recommended with caveats

Note: Scores are presented as standardized percentages (0–100%) calculated according to the AGREE II manual. A score ≥ 60% indicates high quality in that domain. “Strongly Recommended” indicates all domains scored ≥ 60%; “Recommended with caveats” indicates one or more domains scored < 60% but the overall guideline quality is still acceptable. All 3 guidelines met the high-quality threshold in most domains.

#### 3.2.3. Quality evaluation of expert consensus

This study included 2 expert consensus articles, sourced from the CNKI and Wanfang databases.^[22,25]^ Each evaluation criterion was met with a “yes,” indicating a comprehensive design and high quality. Therefore, both articles are recommended. Detailed results are provided in Table 3.

**Table 3:** Quality evaluation results of included expert consensus articles.

Included literature	Are the opinions sourced?	Whether the opinion comes from an influential expert	Is the view centered on the interests of the group?	Is the view logical?	Have you referenced existing literature?	Is the view inconsistent with previous literature?	Overall evaluation
Chinese Society of Cardiothoracic Anesthesiology et al^[22]^	Yes	Yes	Yes	Yes	Yes	No	Recommended
Ji Bingyang et al^[25]^	Yes	Yes	Yes	Yes	Yes	No	Recommended

#### 3.2.4. Quality evaluation of systematic reviews

Among the 2 systematic reviews included in this study, the review by Hla TTW et al^[20]^ was marked as “no” for item 9, “Has the possible publication bias been assessed?.” Additionally, the review by Hasegawa D et al^[19]^ was marked as “unclear” for item 6, “Has the quality evaluation of the literature been completed independently by 2 or more reviewers?.” Despite these issues, the remaining design aspects were thorough, and the overall quality of both reviews was high; thus, both are recommended.

### 3.3. Best evidence summary

This study focused on the anticoagulation management of adult ECMO patients, extracting and synthesizing evidence from the 10 included articles to create an evidence summary. Details are presented in Table 4.

**Table 4:** An integrated summary of the best evidence on anticoagulation management of ECMO in adults.

Evidence dimension	Evidence content	Level of evidence	Recommendation strength
Pre-anticoagulation evaluation			
Identification of high-risk factors	1. It is recommended to identify and correct the potential high-risk factors of ECMO patients before anticoagulation, which can reduce the incidence of bleeding during ECMO operation.^[17]^	2	A
	2. ECMO high-risk factors for anticoagulation: post-cardiac surgery, Hb < 90 g/L, FIB < 2 g/L, PH < 7.12, BMI < 25 kg/m.^[17]^	3	A
	3. In addition to professional knowledge, clinical medical staff are recommended to use a visual coagulation scale as an assessment tool before anticoagulation to identify the patient’s anticoagulation risk.^[23]^	4	B
	4. For patients with severe bleeding or post-cardiac surgery, an anti-Xa factor test should be performed before ECMO anticoagulation to assess the residual heparin in the patient’s body. Protamine can be used to neutralize the residual heparin, and anticoagulation should be performed 24 h after surgery.^[18]^	3	A
Choice of anticoagulant	5. UFH has a rapid anticoagulant effect, is inexpensive, and has a specific antagonist, protamine. It is the preferred anticoagulant method for ECMO, but it is prone to the risk of HIT.^[16]^	2	A
	6. For patients at high risk of heparin-induced HIT, direct thrombin inhibitors can be used instead of UFH anticoagulation, such as argatroban or bivalirudin, but they are expensive and there is no specific antagonist.^[19]^	3	B
	7. Patients with frequent bleeding are allowed to have no anticoagulation, and patients with high bleeding risk can choose low anticoagulation.^[21]^	4	B
Personalized anticoagulation strategy			
Anticoagulation monitoring indicators	8. ACT is currently the most common, simple and rapid monitoring indicator for UFH anticoagulation, but the results may vary due to various factors such as equipment and human operation. Using ACT alone cannot accurately monitor the effects of heparin.^[19]^	2	A
	9. The anticoagulant effect of UFH monitored by APTT is affected by endogenous coagulation factors, so it is recommended to monitor it in combination with ACT to comprehensively judge the anticoagulant effect.^[22]^	2	A
	10. Anti-Xa factor activity detection is the gold standard for adjusting heparin dose and the lowest dose of low molecular weight heparin anticoagulant therapy. It is recommended that ECMO therapy include anti-Xa factor activity detection as part of routine anticoagulation monitoring, with a target range of 0.3–0.7 IU/mL. Bilirubin and plasma free hemoglobin concentrations interfere with the anti-Xa activity test results, but the underlying coagulation state does not affect the results.^[20]^	2	A
	11. Viscoelasticity measurement (ROTEM or TEG) monitoring is not superior to traditional anticoagulation monitoring, but it helps to determine which blood products to take to control bleeding complications.^[17]^	3	A
	12. Optimal anticoagulant therapy (including antithrombin supplementation indications) depends on a comprehensive and standardized evaluation of multiple coagulation indicators such as APTT, anti-Xa, TEG, PLT and FIB.^[24]^	2	A
Prime anticoagulation	13. If time permits, it is recommended to prime the line with fresh frozen plasma or albumin to maintain colloidal osmotic pressure; it is recommended to prime the line with red blood cells, and 50–100 U of heparin can be added for every 1 U of red blood cells.^[17]^	2	A
	14. During emergency ECMO cannulation, Ringer solution can be used for priming, and frozen plasma or platelets can be transfused according to the HCT results.^[18]^	3	A
	15. During ECMO cannulation, after the guidewire is inserted, a loading dose of heparin 1 mg/kg or 50–100 U/kg is given.^[23]^	2	A
Anticoagulation during operation	16. It is routinely recommended to continuously micro-infuse heparin at 5–20 U/kg/h, usually to achieve an anticoagulation target of 20–50 IU/kg/h, and maintain ACT or APTT at 1.5 times the baseline value. For patients with high risk of bleeding, low-intensity anticoagulation is recommended to maintain ACT at 140–160 s or APTT at 40–60 s, and TEG should be monitored when necessary.^[17]^	2	A
	17. It is recommended to monitor factor XIII levels during ECMO operation for patients with coagulopathy. When the XIII level is <70%, it is recommended to supplement factor XIII to reduce the risk of bleeding.^[24]^	4	A
	18. AVWS occurs within 24 h after the start of ECMO operation and requires infusion of cold precipitate or VWF concentrate for treatment, but fibrinogen and coagulation factor VIII need to be monitored to prevent thrombosis.^[23]^	3	A
	19. HIT occurs within 15 min after the start of ECMO operation and lasts until the end. PLT needs to be monitored during anticoagulation to maintain PLT ≧ 50 × 10^9^/L. If HIT is suspected, PLT should be maintained at ≧150 × 10^9^/L. If necessary, coagulation substances should be transfused.^[23]^	2	A
	20. After ECMO combined with PCI, ACT needs to be maintained at 140–220 s during anticoagulation.^[17]^	4	A
	21. During ECMO combined with CRRT treatment, the heparin dose needs to be increased to maintain APTT within the target range.^[17]^	4	A
Complications prevention, treatment and care			
Bleeding	22. When bleeding occurs, the ACT target range can be reduced to 140–160 s. UFH anticoagulation can be suspended for active bleeding. The ECMO speed can be increased to maintain a flow rate of more than 3 L/min. ACT and APTT should be monitored q4h. If necessary, recombinant coagulation factor VIII can be transfused.^[16]^	3	A
	23. Transfusion support: When FIB < 1.0–1.5 g/L, cryoprecipitate and platelets can be transfused to maintain PLT ≧ 50 × 10^9^/L; when INR is 1.5–2.0 and obvious bleeding occurs, fresh frozen plasma can be transfused. When heparin resistance occurs, frozen plasma can be transfused to supplement antithrombin. Infusion of antithrombin concentrate is recommended. If the patient’s bleeding volume is >1000 mL, a large blood transfusion plan can be used. Whole blood transfusion is recommended.^[17,20]^	3	A
	24. Bleeding caused by hyperfibrinolysis can be transfused with fibrinolytic agents, such as 6-aminocaproic acid and tranexamic acid (control the dose to prevent epilepsy).^[22]^	4	B
	25. For refractory hemorrhagic diseases, 40–90 μg/kg rFVIIa is recommended to promote thrombin generation, but caution should be exercised against thrombosis.^[18]^	4	B
Thrombus	26. During ECMO operation, observe all interfaces and monitor the pressure gradient before and after the oxygenator every hour (sudden changes in pressure gradient indicate thrombosis). Use a flashlight to illuminate the model lung every hour to observe whether there is thrombosis in the model lung.^[21]^	2	A
	27. During ECMO operation, maintain a reasonable flow rate, observe whether the speed and flow rate match, whether the tube is shaking, and observe the color of urine. If there is soy sauce urine, it indicates hemolysis. Alkalinization of urine is required and hemodialysis treatment is performed.^[18]^	2	A
	28. When large-area thrombosis or severe hemolysis occurs, the ECMO circuit needs to be replaced and the ECMO treatment time needs to be shortened. The longer the ECMO treatment time, the higher the risk of thrombosis, so it is necessary to be vigilant.^[22]^	3	A
Limb ischemic necrosis	29. During ECMO operation, pay attention to the color, skin temperature, dorsalis pedis artery fluctuations of both limbs, and the circumference of the placed limbs; use B-ultrasound to monitor the blood flow of both limbs.^[25]^	2	A
	30. Assist doctors to establish distal perfusion early to ensure distal blood supply to the lower limbs and prevent ischemic necrosis of the lower limbs.^[23]^	2	A

ACT = activated clotting time, APTT = activated partial thromboplastin time, AVWS = acquired von Willebrand syndrome, BMI = body mass index, CRRT = continuous renal replacement therapy, ECMO = extracorporeal membrane oxygenation, FIB = fibrinogen, Hb = hemoglobin, HCT = hematocrit, HIT = heparin-induced thrombocytopenia, INR = international normalized ratio, PCI = percutaneous coronary intervention, PLT = platelet count, rFVIIa = recombinant activated coagulation factor VIIa, ROTEM = rotational thromboelastometry, TEG = thromboelastography, UFH = unfractionated heparin, VWF = von Willebrand factor.

## 4. Discussion

### 4.1. Evaluation prior to anticoagulation

Evidence 1 to 4 emphasizes the importance of identifying high-risk factors before initiating anticoagulation. The risk of bleeding during ECMO is influenced not only by the ECMO technology itself but also by the patient’s underlying disease conditions. A retrospective study analyzing factors associated with ECMO-related bleeding demonstrated that bleeding within the first 48 hours was independently linked to the patient’s preexisting conditions.^[26]^ Therefore, early identification and correction of potential high-risk factors can significantly enhance treatment efficacy and reduce mortality.

Evidence 5 to 7 addresses the selection of anticoagulants, which are crucial throughout the ECMO treatment process. Choosing the appropriate anticoagulant is essential to prevent thrombosis in the cannula and oxygenator and to minimize bleeding risk. This study provides a comparative analysis of the benefits and drawbacks of unfractionated heparin and thrombin inhibitors. Clinical practitioners must assess the patient’s coagulation status and underlying disease to select the most suitable anticoagulant. Moreover, Evidence 7 suggests that low or no anticoagulation strategies may be viable options. Soltes J et al^[27]^ reviewed the impact of varying anticoagulation intensities on ECMO complications, finding that low-intensity anticoagulation resulted in fewer complications, higher survival rates, and increased safety. Lv et al^[28]^ conducted a meta-analysis comparing low-intensity and standard-intensity anticoagulation in ECMO, revealing that the low-intensity group had significantly lower incidences of gastrointestinal and surgical site bleeding compared to the standard-intensity group. However, clinical data supporting non-anticoagulation approaches remain limited. While non-anticoagulation has been proposed as a potential strategy in specific clinical scenarios, this approach requires further validation through well-designed studies before any definitive conclusions can be drawn. Future research should focus on identifying which patient populations might benefit most from reduced- or no-anticoagulation strategies.

### 4.2. ECMO individualized anticoagulation strategy

Evidence 8 to 12 elucidates the critical importance of monitoring anticoagulation indicators to maintain an optimal anticoagulation level and achieve a balance between anticoagulation and procoagulation during ECMO. Accurate anticoagulation monitoring is essential in this context. Evidence 8 and 9 highlight that activated clotting time and activated partial thromboplastin time are the most commonly used indicators for heparin anticoagulation. However, relying on a single indicator is unreliable due to the potential influence of various confounding factors. Thus, a comprehensive assessment incorporating multiple indicators is necessary to mitigate these influences.

Evidence 10 identifies anti-Xa as a potential gold standard for monitoring heparin anticoagulation, corroborated by prior studies demonstrating its effective anticoagulation properties.^[29]^ Nevertheless, anti-Xa testing fails to account for the roles of platelets and fibrinogen in the coagulation system, and solely depending on this test could overlook bleeding risks associated with coagulation factor deficiencies. Evidence 12 emphasizes that an optimal anticoagulation strategy cannot be formulated based on a single indicator. Instead, it necessitates a comprehensive evaluation using multiple indicators. Consequently, clinical practitioners are advised to employ a combination of monitoring indicators to assess the effects of heparin anticoagulation and the patient’s overall coagulation function.

Evidence 13 to 15 addresses anticoagulation strategies during ECMO priming. Effective anticoagulation should commence during priming to prevent thrombus formation within the circuit. Evidence 16 to 21 outlines specific anticoagulation strategies during ECMO operation, with a primary focus on reducing bleeding risk. This study synthesizes the main causes of bleeding risk during ECMO procedures and offers detailed strategies to guide clinicians in implementing individualized anticoagulation protocols.

### 4.3. Prevention and treatment of complications

Studies indicate a high incidence of complications associated with ECMO anticoagulation, with bleeding occurring in 30 to 50% of cases, thrombosis in approximately 10%, and lower limb ischemia and necrosis in 20%.^[30]^ Evidence 22 to 30 outlines strategies for the prevention, treatment, and care of these common complications. It is imperative for medical staff to engage in meticulous management throughout ECMO treatment. This includes implementing preventive interventions based on abnormal patient indicators, clinical signs, and deviations during ECMO machine operation. Such proactive measures are essential to prevent complications effectively and enhance the safety of ECMO therapy.

## 5. Conclusions

This study comprehensively reviewed and evaluated high-quality literature on ECMO anticoagulation for adults, sourced both domestically and internationally. It consolidated the best available evidence for managing ECMO anticoagulation into a structured summary comprising 3 primary categories, 8 secondary categories, and 30 tertiary categories. This compilation aims to assist clinical practitioners in standardizing ECMO anticoagulation practices based on the most robust evidence. Future research should focus on applying this evidence summary to clinical settings, facilitating the implementation of evidence-based practices, enhancing the safety of ECMO anticoagulation therapy, and preventing associated complications. Additionally, such research will contribute to the development of standardized clinical protocols and procedures.

## Acknowledgments

The authors thank all members of the evidence-based team who participated in the literature quality evaluation and evidence grading. All named individuals have given their written consent to be acknowledged. The authors declare no conflicts of interest.

## Author contributions

**Conceptualization:** Dacai Xu, Weifeng Tang.

**Data curation:** Dacai Xu, Lu Wang, Zheng Li.

**Formal analysis:** Zheng Li.

**Methodology:** Dacai Xu, Lu Wang.

**Project administration:** Weifeng Tang.

**Writing – original draft:** Dacai Xu.

**Writing – review & editing:** Dacai Xu, Weifeng Tang.
